# NOTCH1 Can Initiate NF-κB Activation via Cytosolic Interactions with Components of the T Cell Signalosome

**DOI:** 10.3389/fimmu.2014.00249

**Published:** 2014-05-26

**Authors:** Hyun Mu Shin, Mulualem E. Tilahun, Ok Hyun Cho, Karthik Chandiran, Christina Arieta Kuksin, Shilpa Keerthivasan, Abdul H. Fauq, Todd E. Golde, Lucio Miele, Margot Thome, Barbara A. Osborne, Lisa M. Minter

**Affiliations:** ^1^Program in Molecular and Cellular Biology, University of Massachusetts/Amherst, Amherst, MA, USA; ^2^Department of Veterinary and Animal Sciences, University of Massachusetts/Amherst, Amherst, MA, USA; ^3^Program in Molecular Biology, Loyola University Medical Center, Maywood, IL, USA; ^4^Chemical Synthesis Core Facility, Mayo Clinic, Jacksonville, FL, USA; ^5^Center for Translational Research in Neurodegenerative Disease, University of Florida, Gainesville, FL, USA; ^6^Department of Neuroscience, College of Medicine, University of Florida, Gainesville, FL, USA; ^7^Department of Medicine and Pharmacology, University of Mississippi Medical Center, University of Mississippi Cancer Institute, Jackson, MS, USA; ^8^Department of Biochemistry, Center of Immunity and Infection, University of Lausanne, Epalinges, Switzerland

**Keywords:** NOTCH1, CARMA1, PKCθ, NF-κB, non-canonical, cytosolic, T cell subject category: immunology, signal transduction

## Abstract

T cell stimulation requires the input and integration of external signals. Signaling through the T cell receptor (TCR) is known to induce formation of the membrane-tethered CBM complex, comprising CARMA1, BCL10, and MALT1, which is required for TCR-mediated NF-κB activation. TCR signaling has been shown to activate NOTCH proteins, transmembrane receptors also implicated in NF-κB activation. However, the link between TCR-mediated NOTCH signaling and early events leading to induction of NF-κB activity remains unclear. In this report, we demonstrate a novel cytosolic function for NOTCH1 and show that it is essential to CBM complex formation. Using a model of skin allograft rejection, we show *in vivo* that NOTCH1 acts in the same functional pathway as PKCθ, a T cell-specific kinase important for CBM assembly and classical NF-κB activation. We further demonstrate *in vitro* NOTCH1 associates physically with PKCθ and CARMA1 in the cytosol. Unexpectedly, when NOTCH1 expression was abrogated using RNAi approaches, interactions between CARMA1, BCL10, and MALT1 were lost. This failure in CBM assembly reduced inhibitor of kappa B alpha phosphorylation and diminished NF-κB–DNA binding. Finally, using a luciferase gene reporter assay, we show the intracellular domain of NOTCH1 can initiate robust NF-κB activity in stimulated T cells, even when NOTCH1 is excluded from the nucleus through modifications that restrict it to the cytoplasm or hold it tethered to the membrane. Collectively, these observations provide evidence that NOTCH1 may facilitate early events during T cell activation by nucleating the CBM complex and initiating NF-κB signaling.

## Introduction

Antigen-specific signaling in T cells originates at the membrane *via* the T cell receptor (TCR) and culminates in nuclear transcription of genes that effect specific biological outcomes. This tightly regulated process requires the oligomerization and physical association of CARMA1, BCL10, and MALT1 into the macromolecular CBM complex (1, 2). Successful assembly of the CBM structure requires the upstream actions of kinases such as PDK1 and GLK, which facilitate phosphorylation of PKCθ, and CARMA1, which itself is phosphorylated by PKCθ (3–7). Loss of any of the CBM components stymies full T cell activation (1, 8–10). PKCθ-deficient T cells are unable to form CBM signalosomes and show faulty activation following stimulation through the TCR, including reduced CD25 expression, low levels of IL-2 production, and decreased proliferative potential (6). These defective responses are due in part to insufficient activation of the NF-κB signaling cascade (11).

The NF-κB family of nuclear transcriptional regulators comprises five subunits, p50, p65, c-rel, RelA, and RelB. These subunits co-assemble into homo- or heterodimers to direct unique and specific transcriptional regulation when they bind to recognition elements in the promoters of target genes (12). Two pathways of NF-κB signaling have been described, each of which culminate in distinct biological outcomes. Signaling through CD40–CD40L interactions in T cells can initiate the non-classical NF-κB cascade, while the classical pathway is thought to be the primary mediator of T cell activation down-stream of TCR engagement. This process requires PKCθ phosphorylation, inhibitor of kappa B kinase (IKK) activation, and phosphorylation of its target substrate, inhibitor of kappa B alpha (IκBα), all of which serve to liberate NF-κB subunits from their inactivating complex in the cytosol and initiate classical NF-κB signaling (13, 14). Thus, through its direct action on the CBM complex, PKCθ links proximal TCR signals with temporally delayed biological outcomes mediated by transcription of NF-κB target genes (6).

NOTCH proteins (NOTCH1–4) are evolutionarily conserved transmembrane receptors critically important to an array of biological functions. Mammalian NOTCH binds ligands from one of two families, designated as Delta-like ligand (Dl1, 3, 4) or Jagged (Jag1, 2). In the immune system, NOTCH signaling is vital for T cell development, activation, proliferation, and differentiation into T helper subsets (15). NOTCH receptors undergo a series of enzymatic cleavages, including a final activating cleavage by gamma-secretase which liberates the intracellular, signaling-competent form of NOTCH (NIC) from the cell membrane and allows its translocation to the nucleus. This final cleavage event can be prevented pharmacologically with inhibitors of gamma-secretase (GSI). NOTCH1 signaling is required for peripheral T cell activation and, like PKCθ-deficient T cells, T cells with impaired NOTCH1 signaling show reduced CD25 expression, decreased IL-2 production, and attenuated proliferation (16–18). We previously showed that nuclear NOTCH1 (N1IC) is required to sustain T cell activation and proliferation by retaining NF-κB in the nucleus at time points exceeding 12 h post-stimulation (19). More recently, a novel, extra-nuclear role was attributed to N1IC. That study showed 48 h after stimulation, in regulatory T cells, N1IC uniquely redistributed to the cytosol and associated with cytoplasmic RICTOR to protect regulatory T cells from apoptosis following cytokine withdrawal (20–22). However, a cytosolic function for N1IC in regulating cellular events that occur within minutes to hours after T cell activation has not been fully explored.

Therefore, we focused our inquiry on molecular interactions that occur downstream of TCR engagement and their potential to influence early NF-κB induction in activated T cells. Specifically, we asked whether NOTCH1 might act as a cytosolic scaffold to facilitate CBM assembly and NF-κB activation. Using microscopy and biochemical approaches, we show that cytosolic N1IC associated physically with PKCθ and CARMA1 and nucleated the CBM complex. Blocking NOTCH1 protein expression using shRNA abrogated its association with PKCθ and CARMA1. Surprisingly, in the absence of NOTCH1 expression, the ability of CBM members to bind each other also was lost, suggesting cytosolic NOTCH1 may act as a scaffold protein to facilitate the docking and aggregation of CBM components. Finally, using a luciferase reporter assay and N1IC constructs with restricted localization, either to the cytosol or to the plasma membrane, we demonstrated that non-nuclear N1IC was capable of inducing robust NF-κB activity in stimulated T cells. Altogether, these data support a novel model suggesting N1IC has the potential to function in the cytoplasm to amplify TCR signals and promote early T cell activation through its physical association with members of the CBM complex.

## Materials and Methods

### Animals

All mouse protocols were approved by the Institutional Animal Care and Use Committee of the University of Massachusetts/Amherst. Offspring between the ages of 9 and 12 weeks were used in experiments. PKCθ^null^ mice were obtained from Dan Littman (New York University, NY, USA). C57BL/6, p50^null^, and *NOTCH1* floxed mice were obtained from the Jackson Laboratory (Bar Harbor, ME, USA). *NOTCH1* conditional floxed mice (N1KO) were generated by crossing *NOTCH1^fl/fl^* (*NOTCH1*^tm2Rko^*^/GridJ^*) to *Mx1-Cre*^±^ [B6.Cg-Tg(*Mx1*-cre)1Cgn/J] from the Jackson Laboratory (Bar Harbor, ME, USA). To conditionally delete *NOTCH1* from splenocytes of skin allograft recipients, poly(I)–poly(C) (Amersham Biosciences, Piscataway, NJ, USA) was dissolved in phosphate buffered saline (PBS) and given *in vivo* as previously described (23).

### Murine skin allografting and *in vivo* administration of GSI

Recipient mice (p50^null^, PKCθ^null^, N1KO, WT) were all on a C57BL/6 background. Donor grafts from BALB/c or C57BL/6 mice were prepared by removing the skin from the ventral side of the ear and placing in cold PBS. Recipient mice were anesthetized using isoflurane and administered 5 mg/kg of Ketofen^®^ (Ketoprofen) subcutaneously. Fur was shaved off the dorsal torso of recipients and graft beds were prepared by removing skin from two adjacent sites. Donor skin was placed over the graft bed and excess donor skin from the donor was trimmed away. The allograft was secured using Vaseline^®^ gauze and a Band Aid^®^ which was fastened using silk suture. On day 7, bandages were removed and grafts were visually scored for signs of rejection using a scale of 1–10, with 1 being completely rejected. An allograft was considered fully rejected when it was >80% necrotic (24). Each recipient mouse also received a syngeneic skin graft to control for the integrity of the grafting technique. Some mice were fed GSI in rodent chow (Tekland) formulated to deliver 5 mg/kg/day beginning 2 weeks prior to skin allografting and continuing until grafts were considered to be fully rejected.

### Plasmids

The N1IC parental pcDNA3 expression plasmids were a kind gift of A. Capobianco (25). Mutant constructs were generated by subcloning into pEGFP vector-C1 (BD Biosciences) at the *Bgl*II and *Sal*I sites. The N1IC constructs containing either a nuclear export signal (NES) or a nuclear localization signal (NLS) were previously described (22): **LALKLAGLDL**EQKLISEEDL (NES sequence is in bold and Myc epitope is underlined), **PKKKRKV**EQKLISEEDL (NLS sequence is in bold, and Myc epitope is underlined). cDNAs of N1IC–NES or N1IC–NLS were subcloned into *Bam*HI and *Sal*I sites of pEGFP-C1 vector [BD Biosciences; Ref. (19)]. The cDNA of NOTCH1ΔE from pGD-ΔE was subcloned into *Bgl*II and *Sal*I sites of pEGFP-C1 vector. N1IC in the pEGFP–N1IC–NES construct, was replaced with cDNAs of N1IC mutants to be fused to additional NES, and confirmed by sequencing and by immunoblotting following overexpression in 293T cells. cDNA of CARMA1 in pCR3 expression plasmid with vesicular stomatitis virus (VSV) tag was previously described (26). We constructed the pRRL U6 shRNA PGK puro SIN LTR-containing shRNA sequence against NOTCH1 using the VSV envelope glycoprotein (G protein) expression plasmid (pHCMV-G) and the packaging plasmid for HIV-1-based vectors (pCMVΔR8.2), which is used in lentivirus particle production (kindly provided by S. A. Stewart, Washington University School of Medicine, St. Louis, MO, USA). The reporter construct of NF-κBx3 Luc was purchased from Clontech Laboratories, Inc. (Mountain View, CA, USA). To produce the NOTCH1ΔE-PM construct, site-directed mutagenesis of NOTCH1ΔE was accomplished using the QuikChange mutagenesis system (Stratagene, La Jolla, CA, USA) according to the manufacturer’s protocol. The following primer set was used to introduce point mutations within the cleavage site of Val1744 in NOTCH1: Val to Gly 5′-C TTC GTG GGC TGC GG**T** G*GT* CTG CTG TCC CGC AAG-3′, Val to Gly antisense 5′-CTT GCG GGA CAG CAG **AC**C **A**CC GCA GCC CAC GAA G-3′. Substituted codons are in bold. PCR conditions: 94°C for 30 s, 94°C for 30 s, 55°C for 1 min, and 68°C for 9 min (18 cycles), then followed by the treatment with 1 μl *Dpn*I restriction enzyme at 37°C for 2 h. The ΔE point mutation construct generated by PCR was confirmed by sequencing and by immunoblot following overexpression in 293T cell line.

### Antibodies

Anti-p50 (sc-1190X), anti-c-Rel (sc-71X), anti-p65 (sc-372X), anti-IκBα (sc-371), anti-NOTCH1 (sc-6014-R), anti-GFP (sc-8334), anti-BCL10 (sc-9558 and sc5273), anti-CARMA1 (sc-20458), anti-IKKα (sc-7606), anti-IKKγ (sc-8330), normal goat IgG (sc-2043), and normal rabbit IgG (sc-2027) were purchased from Santa Cruz Biotechnology (Santa Cruz, CA, USA); cleaved N1 (#2421) and phospho-IκBα (#9241) antibodies were from Cell Signaling Technology (Beverly, MA, USA). GAPDH antibody (MAB374) was from Chemicon International, Inc. (Temecula, CA, USA). Anti-human CD3ε (145-2C11) and anti-human CD28 were obtained from R&D Systems (Minneapolis, MN, USA). Anti-CARMA1 (#3189) was obtained from ProSci, Inc. (Poway, CA, USA). Cholera toxin (CTX) B subunit, FITC-labeled (C 1655), monoclonal anti-VSV glycoprotein (V 5507), and anti-β-actin were purchased from Sigma-Aldrich (St. Louis, MO, USA).

### Cell culture

Jurkat T cell lines were cultured in RPMI media containing 10% fetal bovine serum (FBS, Invitrogen), 10 μM β-mercaptoethanol, penicillin/streptomycin, and gentamicin. Sixty millimeter dishes were pre-coated with 20 μg/ml of anti-mouse IgG at room temperature for 2 h and then incubated with 5 μg/ml, each, of anti-human CD3ε and anti-human CD28 at 4°C, overnight. Jurkat T cells (1.5 × 10^7^ cells per dish) were seeded into antibody-pre-coated 60 mm dishes for indicated time periods and harvested for the experiments described below. Peripheral blood mononuclear cells (PBMC) were prepared from buffy coats (LifeSource, Glenview, IL, USA) obtained from healthy adult donors on Ficoll-PAQUE gradient (GE Healthcare, Uppsala, Sweden). For primary human T cell experiments, CD4^+^ T cells were purified from PBMC by negative selection using LS columns (Miltenyi Biotech, Sunnyvale, CA, USA). CD4^+^ T cells were ≥95% pure. Cells were cultivated in 37°C incubator and 5% CO_2_ in RPMI 1640 medium supplemented with 10% FBS (Hyclone, Thermo Fischer Scientific, Waltham, MA, USA), penicillin–streptomycin, 2 mM glutamine, 1 mM pyruvate, 10 mM HEPES, and 0.1 mM non-essential amino acids. CD4^+^ T cells were stimulated by anti-CD3ε and CD28-coated Dynabeads (Invitrogen/Life Technologies, Grand Island, NY, USA), at a 1:1 (bead:cell) ratio.

### Immunological synapse formation – CD4^+^ T cell-bead conjugation

Dynabeads (2 × 10^5^) were coated with anti-CD3ε and CD28 then were combined with primary mouse CD4^+^ T lymphocytes (1:1 ratio) in 200 μl of serum-free media for 5 min at room temperature. The bead-T cell mixtures were then loaded onto a poly-l-lysine-coated glass coverslips for 15–30 min at 37°C, rinsed briefly in PBS, and immediately fixed.

### Immunofluorescence staining and confocal microscopy analysis

Samples were fixed 20 min in 3% paraformaldehyde (PFA) in PBS, quenched with 50 mM NH_4_Cl/PBS, permeabilized for 1 min in 0.3% Triton X-100, and blocked with a solution of PSG [PBS, 0.01% saponin, 0.25% fish skin gelatin, and 0.1% NaN_3_ (all from Sigma-Aldrich)]. The fixed cells were incubated for 1 h with the primary antibodies against PKC-θ and NOTCH1 (Santa Cruz Biotechnology), washed five times in PSG, and then incubated for 1 h with the fluorochrome-labeled rabbit or donkey-anti-species-specific secondary antibodies (Jackson ImmunoResearch, West Grove, PA, USA). The coverslips were then washed five times in PSG, rinsed in ddH_2_O, and mounted with ProLong^®^ Gold Antifade reagent (Invitrogen/Life Technologies). Stained cells were visualized with a Zeiss LSM 510 Meta Confocal Microscope, using a 63× oil immersion objective. Thirty-five to 50 z-sections, separated by 0.2 μM, were acquired.

### Lipid raft isolation

Jurkat T cells (5 × 10^7^ cells) were stimulated as described and lysed on ice for 20 min in 500 μl of 1% Triton X-100 in MN buffer (25 mM MES, 150 mM NaCl, pH 6.5) supplemented with protease inhibitor cocktail (Sigma-Aldrich). The cell lysate was homogenized with a loose-fitting Dounce homogenizer (15 strokes) and spun at 500 × *g* for 7 min at 4°C. Post-nuclear supernatant (400 μl) was mixed with 400 μl of 80% sucrose diluted with MN buffer, then overlaid with 800 μl of 36% sucrose and 400 μl of 5% sucrose. The gradients were spun for 24 h at 4°C at 220,000 × *g*. Nine fractions (220 μl each) were collected from bottom to top. All fractions were analyzed by dot blot with anti-CTX-conjugated horse radish peroxidase (HRP) prior to immunoblotting with the antibodies indicated in each figure.

### Co-immunoprecipitation assays and western blotting

293T cells were transfected with the plasmids indicated, using FuGENE6^®^ (Roche Diagnostics, Indianapolis, IN, USA). After 2 days, cells were lysed for 30 min at 4°C in 500 μl of 1% NP-40 lysis buffer (10 mM Tris-HCl pH 7.8, 0.5 mM EDTA, 250 mM NaCl, and protease inhibitor cocktail). For Jurkat T cells, 1.5 × 10^7^ cells were incubated in 60 mm dishes pre-coated, as described, with anti-human CD3ε and anti-human CD28 for the indicated time points, then lysed in 500 μl of 1% NP-40 lysis buffer. Supernatants were incubated with 10 μl of normal serum and 100 μl of protein G-sepharose beads (Pharmacia, Stockholm, Sweden) at 4°C on a rotator for pre-clearing. After centrifugation, pre-cleared supernatants were incubated at 4°C on a rotator, overnight, with 2 μg of each antibody, as indicated in figures. For co-immunoprecipitated samples, goat normal IgG or rabbit normal IgG (Santa Cruz Biotechnology) served as negative controls. Seventy microliters of protein G-sepharose beads were then added and incubated for an additional 60 min at 4°C on a rotator. The beads were washed five times with 1% NP-40 lysis buffer containing 10 mM Tris–HCl pH 7.8, 0.5 mM EDTA, 250 mM NaCl with protease inhibitor cocktail, then boiled in Laemmli buffer and assayed by immunoblot. Human primary CD4^+^ T cells were lysed in 200 μl of mammalian protein extraction reagent (M-PER; Pierce Biotechnology, Rockford, IL, USA) containing protease inhibitors (Roche Diagnostics) and Halt™ phosphatase inhibitors (Thermo Fischer Scientific). Thirty micrograms of protein was loaded for western blotting. Band intensity was determined using ImageJ Software (NIH).

### Gel shift mobility assays (EMSA)

The NF-κB consensus binding site oligo was labeled with ^32^P following the manufacturer’s instructions (Promega, Madison, WI, USA). Briefly, 2 μl of the oligo (at 1.75 pmol/ml concentration) was incubated with 1 μl of 10× T4 polynucleotide kinase buffer (700 mM Tris–HCl, pH 7.6, 100 mM MgCl_2_, 50 mM DTT), 1 μl of ^32^P γATP (3000 Ci/mmol at 10 mCi/ml), 5 μl of autoclaved dH_2_O, and 1 μl of T4 polynucleotide kinase (5–10 μ/ml) at 37°C for 10 min. The reaction was stopped by adding 1 μl of 0.5 M EDTA and the total volume was brought up to 100 μl by adding 89 μl of TE buffer (10 mM Tris–HCl, pH 8.0, 1 mM EDTA). The labeled oligo was carefully applied to the center of the column bed of Sephadex G-50 column and centrifuged at 1100 × *g* for 4 min and radioactivity was assessed using a liquid scintillation counter. About 5 μg of nuclear extracts were incubated with ^32^P-labeled NF-κB probes for 20 min at room temperature. The reactions were generally in 9 μl total volume containing 2 μl of 5× gel shift-binding buffer [20% glycerol, 5 mM MgCl_2_, 2.5 mM EDTA, 2.5 mM DTT, 250 mM NaCl, 50 mM Tris–HCl, pH 7.5, 0.25 mg/ml poly (dI–dC)–poly (dI–dC)], nuclear extract, and nuclease-free water. For super-shift assays, reactions were incubated for 2 h in the presence of 2 μg of indicated antibodies (Santa Cruz Biotechnology). When labeled oligo was competed with excess same (NF-κB) or unrelated (SP-1) unlabeled oligo, 1 μl of unlabeled oligo was added to the reaction and water volume was adjusted accordingly to keep the reaction at 9 μl total volume. The reaction mix was incubated for 10 min at room temperature and then for another 20 min once the labeled oligo was added. The NF-κB and SP-1 consensus oligonucleotides were purchased from Promega. The DNA–protein complexes were then mixed with 1 μl of 10× gel loading buffer (250 mM Tris–HCl pH 7.5, 0.2% bromophenol blue, 40% glycerol) and subjected to electrophoresis in 4% acrylamide gels at 250 V at room temperature in cold 0.5× TBE buffer (0.5 M Tris base, 0.4 M boric acid, 0.005 M EDTA, pH 8.0). Before loading the gels with samples, the gels were pre-run for 30 min at 250 V at room temperature. The gels were dried and subjected to autoradiography.

### Dual-luciferase reporter gene assay

Jurkat T cells were plated on 60 mm dishes and transfected with the indicated expression vectors or with empty vector, as a control. In the different experiments, we used 0.4 μg of NF-κBx3 luc as the reporter plasmid and 0.1 μg pRL-CMV as the internal control. Luciferase assays (Dual-Luciferase Assay System, Promega) were performed 48 h after transfection, according to the manufacturer’s instructions. Before harvest, transfectants were stimulated for 3 h with phorbol 12-myristate 13-acetate (PMA) and calcium ionophore (CaI) both from Sigma-Aldrich at 80 and 500 nM, respectively. Luciferase values were normalized against Renilla luciferase activity. A minimum of three independent experiments were performed, in duplicate.

### Lentiviral transduction with *NOTCH1* shRNA

293T cells were seeded in 100 mm culture dishes (3 × 10^6^ per dish) 1 day prior to transfection. The transfection mixture complexes were prepared by mixing together 100 μl serum-reduced OPTI-MEM (Invitrogen), 36 μl FuGENE6^®^ (Roche Diagnostics), 12 μg of DNA (6 μg pRRL-shNOTCH1, 3 μg pHCMV-G, and 3 μg pCMVΔR8.2) and incubating at room temperature for 20 min. The transfection mixture was added to 1 ml complete media and then transferred onto 293T cells. After 24 h of incubation, DNA-transfection medium was replaced with fresh culture medium. The lentiviral culture supernatants were harvested 48 h post-transfection. Jurkat T cells were plated for lentiviral infection at 1 × 10^7^ per 100 mm dish in 1 ml of culture media, 10 ml of 0.4 μm filtered lentiviral culture supernatants, and 10 μg/ml polybrene. After 24 h of infection with lentiviral supernatant, 20 ml of fresh RPMI media containing 10% FBS, 10 μM β-mercaptoethanol, penicillin/streptomycin, gentamicin, and 0.5 μg/ml of puromycin was added and cells were and selected for 72 h post-infection. Cells were assayed for successful reduction of endogenous expression of NOTCH1 by immunoblotting. For human PBMCs, cells were cultivated in 37°C incubator and 5% CO_2_ in RPMI 1640 medium supplemented with 10% FBS (Hyclone), penicillin–streptomycin, 2 mM glutamine, 1 mM pyruvate, 10 mM HEPES, and 0.1 mM non-essential amino acids. CD4^+^ T cells were stimulated by anti-CD3ε and CD28-coated beads (Invitrogen/Life Technologies) using one bead per cell. For shRNA experiments, freshly isolated CD4^+^T cells were transfected with scrambled control shRNA or NOTCH1 shRNA (Santa Cruz Biotechnology) using the Amaxa Nucleoporator system according to manufacturer’s instructions. CD4^+^ cells (5–10 × 10^6^) were resuspended in 100 μl of nucleofection solution and transfected with 100 nM shRNA using U-014 nucleofector program (Lonza Group, Worldwide). After transfection, cells were incubated for 6 h at 37°C then fresh medium was added. After 24 h, transfected cells were stimulated with anti-CD3ε and CD28-coated beads (Invitrogen/Life Technologies). Trypan blue exclusion assay was performed to ensure cells’ viability after nucleoporation.

### Statistical analyses

Data are represented as means + SEM. Analyses were performed using Prism software (GraphPad) with a one-way ANOVA with Tukey’s post-test applied. *P* < 0.05 was considered significant.

## Results

### NOTCH1 and PKCθ act within the same functional pathway to mediate skin allograft rejection

T cell receptor engagement together with co-stimulation through CD28 will fully activate peripheral T cells, an event that requires phosphorylation of the T cell-specific kinase, PKCθ. T cells from transgenic mice deficient in PKCθ do not exhibit a characteristic activation phenotype. Instead, they fail to upregulate CD25, the high-affinity IL-2 receptor, exhibit faulty NF-κB activity, and show reduced levels of IL-2 production (1). PKCθ has been previously described to play an essential role in activating the classical NF-κB signaling cascade (14). Interestingly, when peripheral T cells deficient in NOTCH1 signaling are stimulated through the TCR, they exhibit a similar phenotype of dampened T cell activation (16–19, 23). This observation led us to ask whether PKCθ and NOTCH1 cooperate within the same signaling cascade to direct full T cell activation and subsequent downstream effects.

**Figure 1:**
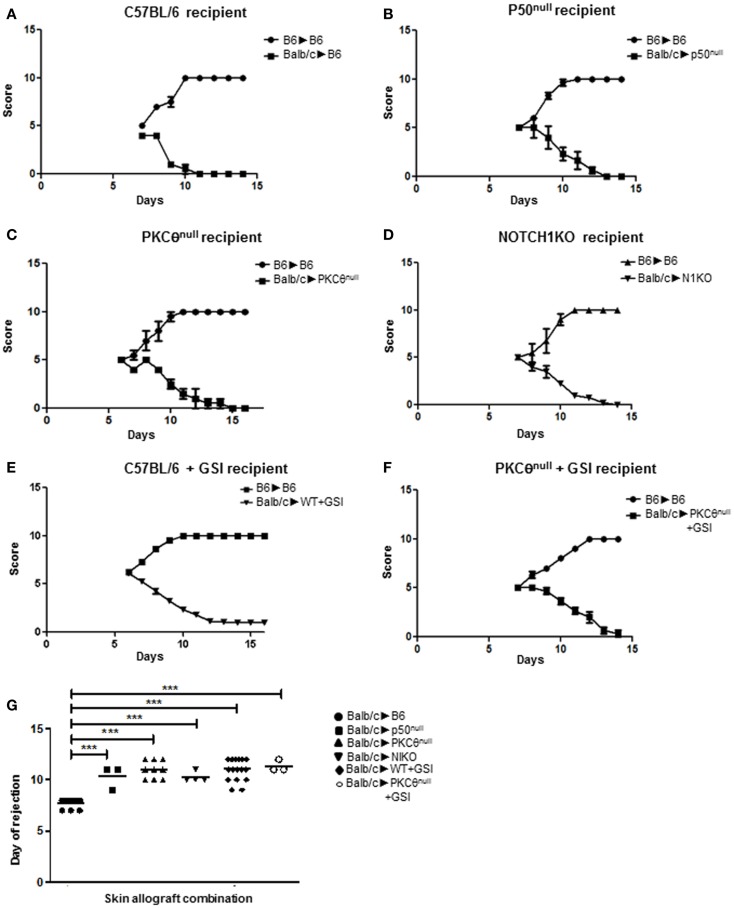
**NOTCH1 and PKCθ act in the same functional pathway to mediate allograft rejection**. We monitored the influence on rejection kinetics of murine skin allografts by recipient mice deficient for various proteins. Grafts were visually scored for signs of rejection using a scale of 1–10, with 1 being completely rejected. An allograft was considered fully rejected when it was >80% necrotic. Graphs represent allograft score (*y* axis), with a score of “1” being fully rejected and a score of “10” being fully accepted *versus* time in days after skin grafting (*x* axis). Wild-type BALB/c skin was grafted onto **(A)** wild-type C57BL/6 mice (BALB/c → BL6), which served as a baseline for graft rejection for subsequent allograft combinations; **(B)** p50^null^ mice (BALB/c → p50^null^); **(C)** PKCθ^null^ mice (BALB/c → PKCθ^null^); **(D)** NOTCH1 conditional knock-out (N1KO) mice (BALB/c → N1KO); **(E)** wild-type C57BL/6 mice whose NOTCH signaling was abrogated by administering γ-secretase inhibitor (GSI LY411,575; BALB/c → WT + GSI); and **(F)** PKCθ^null^ mice whose NOTCH signaling was abrogated by administering γ-secretase inhibitor (GSI LY411,575) in chow (BALB/c → PKCθ^null^ + GSI). **(G)** Day to complete rejection was compared between different groups of recipient mice. For each animal grafted with BALB/c skin, an internal control of C57BL/6 skin was also grafted (BL6 → BL6) to monitor integrity of the grafting technique. Data represent the mean + SEM (*n* = 3–16 mice/group). ***P < 0.001; one-way ANOVA with Tukey’s post-test applied.

We tested this functional interaction, *in vivo*, using a murine model of skin allograft rejection. Full acceptance of BALB/c cardiac allografts can be induced in C57BL/6 recipients when CD4^+^ T cell-intrinsic, classical NF-κB signaling is inhibited. By contrast, BALB/c skin allografts are fully rejected, albeit with delayed kinetics, when transplanted onto C57BL/6 mice displaying deficient classical NF-κB activity (27). Since we hypothesized that NOTCH1 and PKCθ may be operating within the same cascade to facilitate NF-κB activation, we predicted that inhibiting the activities of either or both proteins in recipient mice would delay skin allograft rejection in this transplantation model, but would not prevent it fully. We established our baseline time to full rejection by transferring skin from a BALB/c donor onto a wild-type C57BL/6 recipient (Figure 1A). When skin from a BALB/c donor was grafted onto C57BL/6 mice deficient in the p50 subunit (p50^null^), which is required for classical NF-κB activation, graft rejection was significantly delayed compared to controls (Figures 1B,G). We used this second point of reference as the average time to rejection when classical NF-κB signaling in recipient mice is impaired. We next assessed how the loss of PKCθ affected graft rejection by transplanting skin allografts from BALB/c mice onto PKCθ^null^ C57BL/6 recipients. The delay in allograft rejection was identical to that seen in p50^null^ allograft recipients (Figures 1B,C,G), confirming that inhibiting either PKCθ or p50 produce identical kinetics of skin graft rejection. We then determined what effect inhibiting NOTCH signaling had on skin allograft survival in this model. We genetically deleted NOTCH1 in peripheral immune cells of *NOTCH1^fl/fl^* recipient mice using cre-lox technology (23), or we inhibited NOTCH activity, pharmacologically, by treating recipient mice with a GSI, to prevent the final cleavage and nuclear translocation of NOTCH receptors. We then transplanted skin from BALB/c donors and followed the time to rejection. Our results show that mice deficient in NOTCH1 signaling (N1KO or +GSI) exhibited the same rejection kinetics as seen with the p50^null^ or the PKCθ^null^ allograft recipients (Figures 1D,E,G). We reasoned that if NOTCH1 and PKCθ function in the same signaling cascade, we would still observe graft rejection, even if both proteins were simultaneously inhibited. To test this hypothesis, we blocked NOTCH signaling in PKCθ^null^ recipient mice by administering GSI formulated in rodent chow then grafted BALB/c skin onto these NOTCH-inhibited, PKCθ^null^ mice. As shown in Figures 1C,F,G, PKCθ^null^ mice show comparable kinetics of graft rejection regardless of whether NOTCH1 signaling was inhibited using GSI chow, providing evidence that NOTCH and PKCθ may operate in the same functional pathway in a murine model of skin allograft rejection.

### NOTCH associates with PKCθ in activated T cells

In regulatory T cells, intracellular NOTCH1 (N1IC) has been shown to physically associate in the cytosol with RICTOR to potentiate cell survival during cytokine withdrawal (20–22). An intriguing extension of these data would propose that sub-cellular localization may be one means by which NOTCH1 mediates its diverse effects during T cell activation. Motivated by our *in vivo* observations, we went on to explore the possibility that PKCθ and NOTCH1 physically cooperate in a T cell-intrinsic fashion to direct full T cell activation.

**Figure 2:**
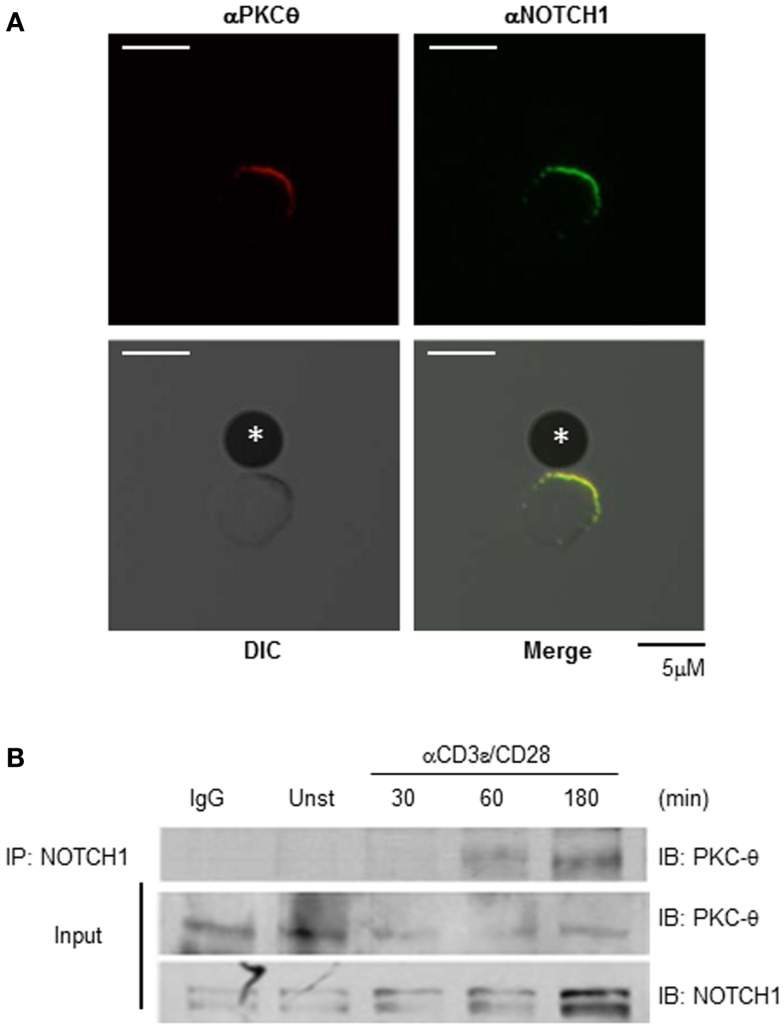
**NOTCH1 associates with PKCθ in stimulated T cells**. NOTCH1 and PKCθ co-localize and interact following T cell stimulation. **(A)** Purified CD4^+^ T cells from C57BL/6 mice were incubated for 30 min with Dynal beads pre-coated with anti-mouse CD3ε and anti-mouse CD28 on glass slides. Cells were fixed, quenched, permeabilized, blocked, and stained with antibodies to NOTCH1 (green) and PKCθ (red). Proteins were visualized using species-specific, fluorescently conjugated secondary antibodies. *Indicates bead. **(B)** Jurkat T cells were stimulated with plate-bound anti-human CD3ε and anti-human CD28 for the indicated time periods, and subjected to co-immunoprecipitation with anti-NOTCH1. Immunoprecipitates (upper panel) and 1/100 of input (lower panels) were immunoblotted with anti-PKCθ and anti-NOTCH1. Data are representative of at least three independent experiments.

To address this question, we incubated WT CD4^+^ T cells from C57BL/6 mice for 15–30 min with Dynal beads pre-coated with anti-mouse CD3ε and anti-mouse CD28. We then stained cells with antibodies specific for NOTCH1 and PKCθ and used confocal microscopy to examine their sub-cellular localization. As shown in Figure 2A, NOTCH1 and PKCθ co-localize at the bead–cell interface, placing them together temporally and spatially within minutes of T cell activation. We next used biochemical methods to assess the physical interaction between NOTCH1 and PKCθ in activated human T cells. Utilizing immunoprecipitation, we could detect NOTCH1 bound to PKCθ as early as 1 h after stimulating human Jurkat T cells with anti-human CD3ε and anti-human CD28, and this association increased in intensity by 3 h (Figure 2B; Figure S1 in Supplementary Material). Cleaved NOTCH1 drives its own expression (28), and we previously determined that NOTCH1^IC^ can be detected in T cells 6–8 h following stimulation (Osborne, unpublished). This expression requires new protein synthesis, since pulsing cells with cycloheximide prevents NOTCH1^IC^ accumulation (data not shown). However, the rapid kinetics of co-localization and complex formation we detected by our combined microscopy and biochemical approaches indicate that the NOTCH1–PKCθ complexes we detected within an hour of stimulation result from the association of pre-existent proteins expressed in T cells at the time of TCR activation.

### NOTCH1 physically interacts with components of the CBM

**Figure 3:**
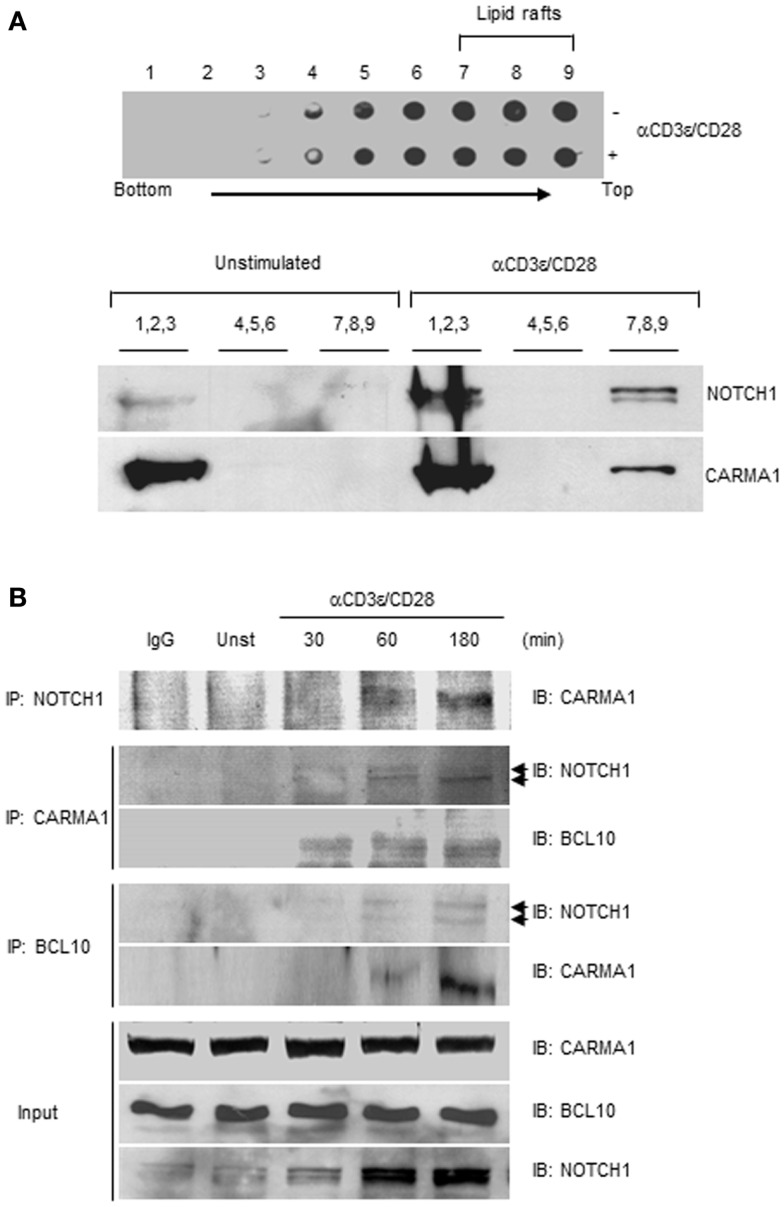
**NOTCH1 interacts with CARMA1 and BCL10 following stimulation with anti-CD3ε and anti-CD28**. **(A)** Jurkat T cells were stimulated with plate-bound anti-human CD3ε and anti-human CD28 and subjected to sucrose gradient centrifugation. All fractions were analyzed by dot blot with anti-cholera toxin-conjugated HRP (upper panel). Pooled fractions were immunoblotted with indicated antibodies (lower panel). **(B)** Jurkat T cells were stimulated as in **(A)** for the indicated time periods, followed by co-immunoprecipitation with antibodies specific for NOTCH1, CARMA1, or BCL10. Eluates were subjected to immunoblotting with indicated antibodies. 1/100 of input (shown) was immunoblotted with anti-CARMA1, anti-BCL10, and anti-NOTCH1. Arrow heads beside IB: NOTCH1 represent 120 kDa, transmembrane form (upper arrow head) and 110 kDa intracellular form (lower arrow head) of NOTCH1. Data are representative of at least three independent experiments.

Following TCR–CD28 stimulation, PKCθ translocates to the site of TCR clustering known as the immunological synapse (IS) and triggers the recruitment of CARMA1 and BCL10 to lipid rafts (29, 30). NOTCH1 has also been shown to localize to the IS and to associate with PI3K and p56^Lck^ in activated T cells at the site of TCR clustering (31). Collectively, these observations place PKCθ and NOTCH1 in the same cellular location as the CBM complex during T cell activation. Since studies show PKCθ associates with and phosphorylates CARMA1 (1, 3), we further explored the possibility that NOTCH1 also interacts physically with CARMA1. To do so we used Jurkat human T cells, which endogenously express NOTCH1 together with signaling components necessary for CBM complex assembly. Lipid rafts were isolated by ultracentrifugation over a discontinuous sucrose gradient from lysates of Jurkat T cells. Nine fractions, in total, were collected and all were tested in dot blots with anti-CTX conjugated with HRP. The 12,000 Da, non-toxic B subunit of CTX binds specifically to the pentasaccharide moiety of ganglioside GM1, a glycosphingolipid predominantly associated with lipid rafts (31, 32). As shown in Figure 3A, upper panel, further analysis by immunoblotting revealed that the lipid raft marker GM1 was exclusively detected in the lipid raft-containing, higher-numbered fractions (No. 4–9), whereas the lower-numbered fractions (No. 1–3) which contain soluble membrane proteins, showed undetectable levels of sphingolipid GM1. When the fractions indicated were pooled and immunoblotted with antibodies specific for NOTCH1 or CARMA1 we observed, in unstimulated Jurkat T cells, the majority of CARMA1 co-localized with NOTCH1 in the soluble fractions (Figure 3A, lower panels, No. 1–3). However, in fractions obtained from Jurkat T cells stimulated with anti-human CD3ε and anti-human CD28, a portion of CARMA1 could be found in the lipid raft fractions (Figure 3A, lower panels, No. 7–9), where it also co-localized with NOTCH1. To determine whether this redistribution involved direct interaction between these molecules, Jurkat T cells were stimulated with antibodies for the indicated time periods, harvested, and whole cell lysates were co-immunoprecipitated using antibodies specific for NOTCH1, CARMA1, or BCL10. Consistent with earlier reports (26), CARMA1 co-immunoprecipitated with BCL10 in stimulated Jurkat T cells (Figure 3B; Figure S2C in Supplementary Material). In addition to binding BCL10, CARMA1 also associated strongly with NOTCH1 (Figure 3B; Figures S2A,B in Supplementary Material), regardless of which protein was the target of the immunoprecipitating antibody. We further confirmed NOTCH1- and CARMA1-interactions with BCL10 by co-immunoprecipitating with anti-BCL10 (Figure 3B; Figures S2D,E in Supplementary Material). Intriguingly, CARMA1 was associated both with membrane-bound NOTCH1 (~120 kDa, *upper arrowhead*) and the intracellular domain of NOTCH1 (~110 kDa, *lower arrowhead*) as shown in Figure 3B, with CARMA1 showing increasing binding over time to the intracellular domain of NOTCH1^IC^.

**Figure 4:**
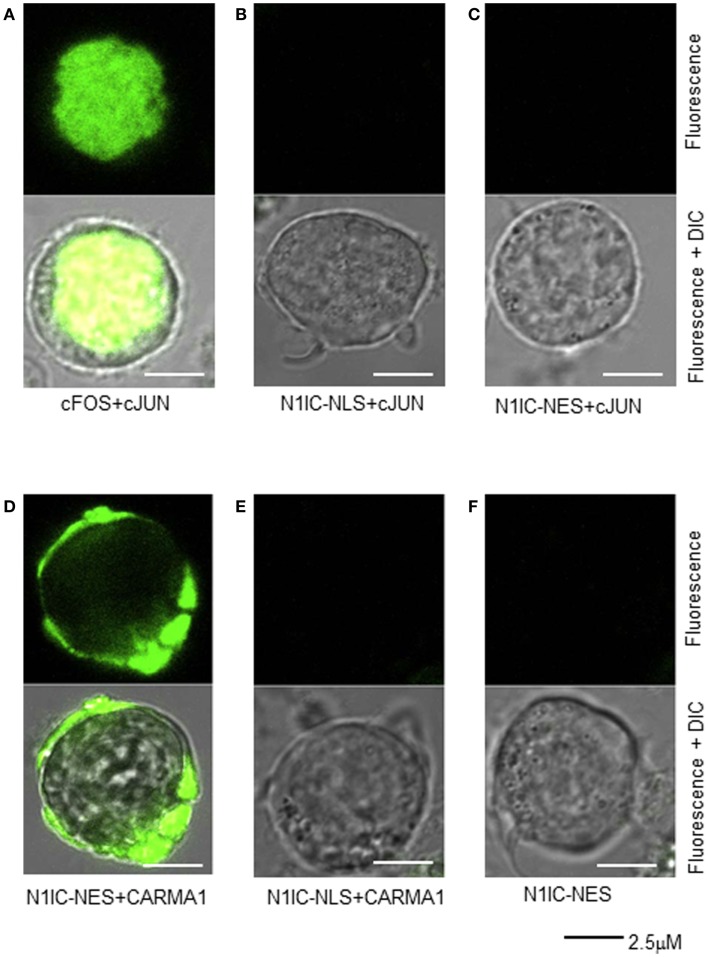
**Cytosolic N1IC can interact with CARMA1 in a bifluorescence complementation assay**. Jurkat T cells were transfected with various constructs of nuclear and/or cytosolic proteins and their physical association was assessed microscopically by their ability to reconstitute two halves of a yellow fluorescent protein reporter. Twenty-four hours after transfection, cells were plated onto glass bottom culture dishes, stimulated with anti-human CD3ε and anti-human CD28, and fluorescent images of live cells were captured using a Zeiss LSM 510 confocal microscope. **(A)** Nuclear proteins, cFos and cJun, known to interact in the nucleus were co-expressed as a control; **(B)** NOTCH1 with an additional nuclear localization signal, N1IC–NLS was co-expressed with nuclear cJun; **(C)** NOTCH1 with an additional nuclear export signal, N1IC–NES, was co-expressed with nuclear cJun; **(D)** NOTCH1 with an additional nuclear export signal, N1IC–NES was co-expressed with CARMA1, a cytosolic protein; **(E)** NOTCH1 with an additional nuclear localization signal, N1IC–NLS was CARMA1; **(F)** NOTCH1 with an additional nuclear export signal, N1IC–NES was expressed alone. Upper panel of all images represent fluorescent channel only; lower panel of all images represent merged fluorescent and DIC images. Data are representative of at least three separate experiments.

Although a cytosolic role for NIC1 has been reported in regulatory T cells (20–22), no such evidence has yet been offered in support of a cytosolic function for N1IC during normal T cell activation. Therefore, we sought to further confirm NOTCH1–CARMA1 association in the cytoplasm of T cells using an alternate approach. Various means exist for determining protein–protein interactions including fluorescence resonance electron transfer (FRET) and enzyme fragment complementation methods (33, 34). More recently, bimolecular fluorescence complementation (BiFC) assays have been developed to interrogate physical interaction between proteins (35, 36). In this approach, a whole fluorescent reporter molecule is expressed as two complementary fragments, each in an individual plasmid, and each linked to one of two proteins of interest. When both plasmids are co-transfected into a cell line, the fluorescent reporter molecule will be reconstituted only if the two proteins of interest physically associate. We validated our approach using BiFC in Jurkat T cells, by co-expressing plasmids containing cFOS (pBiFC–cFos–YC155) and cJUN (pBiFC–cJun–YN155), whose interaction in the nucleus is well-documented. As shown in Figure 4A, co-expression of cFOS and cJUN results in fluorescence complementation and its detection in the nucleus. No fluorescence was detected in the nucleus when pBiFC–cJun–YC155 was expressed together with a plasmid encoding NOTCH1^IC^ that was targeted either to the nucleus (pBiFC–N1IC–*NLS*–YC155; Figure 4B) or to the cytosol (pBiFC–N1IC–*NES*–YC155; Figure 4C) by the addition of a NLS or a NES, respectively. We next transfected Jurkat T cells with the following combinations of constructs: (i) pBiFC–N1IC–*NES*–YC155 and pBiFC–CARMA1–YN155, (ii) BiFC–N1IC–*NLS*–YC155 and pBiFC–CARMA1–YN155, or with (iii) pBiFC–N1IC–*NES*–YC155 alone, to ask whether CARMA1 and NOTCH1 are capable of binding to each other in the cytosol. Twenty-four hours after transfection, cells were incubated with anti-human CD3ε and anti-human CD28. Receptor-bound antibody was cross-linked using anti-goat IgG and fluorescent images of live Jurkat T cells were acquired using confocal microscopy. As shown in Figure 4D, fluorescence was reconstituted in cells which expressed CARMA1 together with the cytoplasmically restricted NOTCH1 (pBiFC–N1IC–*NES*–YC155), but not in cells expressing CARMA1 and the nuclear-targeted NOTCH1 construct (pBiFC–N1IC–*NLS*–YC155; Figure 4E) or in cells expressing pBiFC–N1IC–*NES*–YC155 in the absence of pBiFC–CARMA1–YN155 (Figure 4F). Therefore, using two distinct approaches to interrogate their physical interaction in human T cells, we show for the first time that, following anti-human CD3ε and anti-human CD28 stimulation, NOTCH1 can associate with CARMA1, a component of the cytoplasmically restricted, CBM complex.

### NOTCH1 influences the formation of the CBM complex

**Figure 5:**
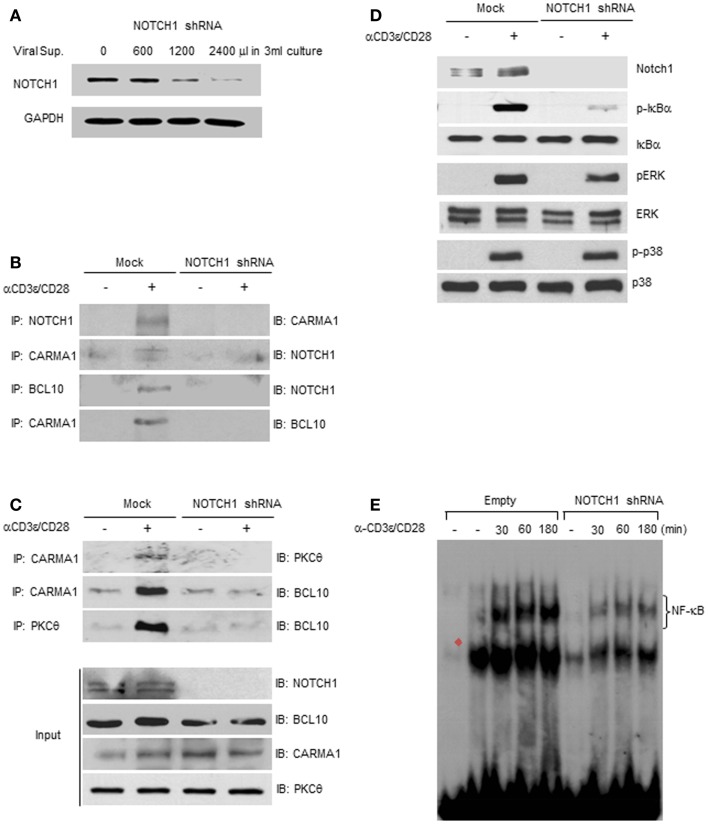
**NOTCH1 deficiency prevents the formation of the CBM complex**. **(A)** NOTCH1 expression was efficiently reduced when Jurkat T cells were incubated with viral supernatants. NOTCH1-knock-down Jurkat T cells, or mock-infected control cells, were stimulated with plate-bound anti-human CD3ε and anti-human CD28 for 1 h, harvested for co-immunoprecipitation with indicated antibodies, then subjected to immunoblotting with **(B,C)** anti-NOTCH1, anti-CARMA1, anti-BCL10, or anti-PKCθ; 1/100 of input was immunoblotted with indicated antibodies, or with **(D)** antibodies specific for NOTCH1, phosphorylated and total IκBα, phosphorylated and total ERK, and phosphorylated and total p38 MAPK. **(E)** NOTCH1-knock-down Jurkat T cells, or mock-infected control cells, were stimulated with plate-bound anti-human CD3ε and anti-human CD28 for the times indicated. EMSAs were performed on nuclear extracts using radiolabeled oligonucleotides containing the NF-κB binding sequence. Red filled diamond represents competition with cold probe. Data are representative of at least three independent experiments.

CARMA1 co-precipitates with the TCR, and is recruited to lipid rafts enriched in PKCθ (26). CARMA1 phosphorylation is likely mediated by PKCθ, following its own phosphorylation by GLK (1, 2, 4, 7). Once phosphorylated, CARMA1 associates with the downstream scaffold/adapter molecules, BCL10 and MALT1, recruiting these molecules into the lipid rafts of the IS and, ultimately, leading to activation of NF-κB (30). Although we could demonstrate that NOTCH1 physically interacts with CARMA1 and BCL10 (Figures 3 and 4), it remained unclear whether this association was necessary for, or was the result of, CBM complex formation. To ask whether NOTCH1 was required for the assembly of the CBM complex, we infected Jurkat T cells with lentiviral constructs corresponding to shRNA to NOTCH1 (NOTCH1-knock-down Jurkat). These cells exhibited reduced NOTCH1 expression inversely proportional to the concentration of lentiviral supernatant in the culture media (Figure 5A). To test whether reducing the level of endogenous NOTCH1 abrogated CBM complex formation, whole cell lysates from NOTCH1-knock-down or mock-infected Jurkat T cells were immunoprecipitated with antibodies specific for NOTCH1, CARMA1, or BCL10, then immunoblotted with antibodies specific for CARMA1 or BCL10 (Figure 5B; Figures S3A–D in Supplementary Material). In the absence of NOTCH1, CARMA1 failed to associate with BCL10, compared to mock-infected controls, indicating that NOTCH1 is indispensable for the interaction between CARMA1 and BCL10.

To test whether the deficiency of endogenous NOTCH1 prevents association of PKCθ with the CBM complex, NOTCH1-knock-down Jurkat T cells or mock-infected controls were stimulated with plate-bound anti-human CD3ε and anti-human CD28. T cell lysates were then co-immunoprecipitated with antibodies specific for CARMA1 or PKCθ. As shown in Figure 5C and in Figures S4A–C in Supplementary Material, CARMA1 interacts both with PKCθ and BCL10 in the mock-infected controls. In contrast, NOTCH1 deficiency resulted in greatly diminished interactions between CARMA1 and PKCθ, as well as between PKCθ and BCL10, even following anti-human CD3ε and anti-human CD28 co-stimulation. To demonstrate that NOTCH1 shRNA does not inhibit all signaling events downstream of TCR activation, we examined the phosphorylation pattern of additional molecules known to be differentially phosphorylated following TCR activation. In Jurkat T cells with NOTCH1-knock-down, as expected, we observed decreased expression of phosphorylated ERK compared to mock-infected controls. However, phosphorylation of p38 MAPK remained unchanged following stimulation with anti-human CD3ε and anti-human CD28 (Figure 5D; Figures S5A,D–G in Supplementary Material). Additionally, and consistent with our previous results, phosphorylation of IκBα was significantly decreased in Jurkat cells transfected with NOTCH1 shRNA, while the level of total IκBα remained unchanged (Figures S5B,C in Supplementary Material). Functionally, this translated into a diminished NF-κB–DNA binding capacity, as compared to mock-infected controls, when DNA binding was measured using an electrophoretic mobility shift assay (Figure 5E). Thus, inhibiting NOTCH1 expression using shRNA approaches had a significant effect on phosphorylation of IκBα, a critical step in the NF-κB activation cascade, as well as functionally, on NF-κB–DNA interactions, but showed little or no effect on other targets of the TCR signaling pathway such as phosphorylation of p38. Taken together, these data suggest that NOTCH1 may influence early NF-κB activation in stimulated T cells through its function as a scaffold protein and its potential recruitment by PKCθ to the CBM complex, upstream of IKK activation and IκBα phosphorylation.

### Functional domains of N1IC mediate its interaction with CARMA1

**Figure 6:**
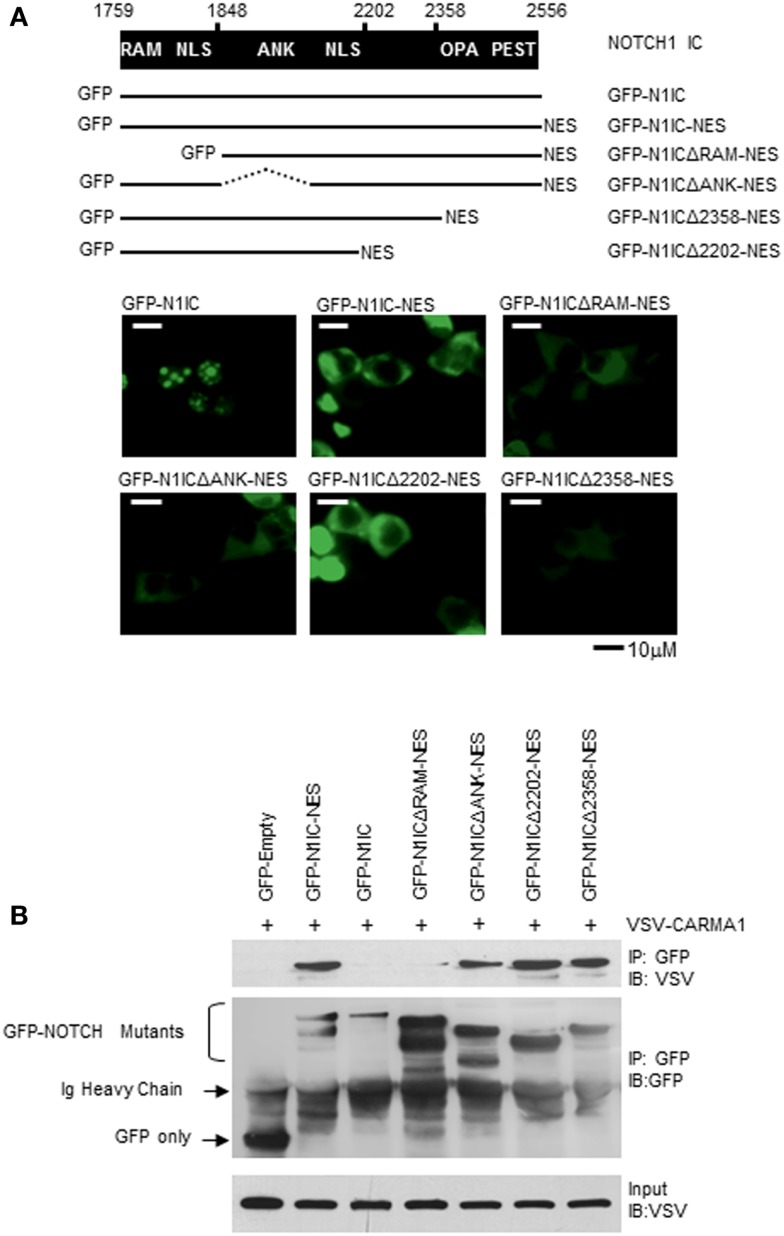
**The RAM domain of NOTCH1 mediates its interaction with CARMA1**. **(A)** GFP–N1IC mutant expression plasmids were transfected into 293T cells and sub-cellular localization of NOTCH1 was examined using fluorescence microscopy. **(B)** VSV–CARMA1 expression plasmids were transiently co-transfected with GFP empty vector as a control, or with GFP–N1IC mutant plasmids in 293T cells, as indicated. Transfected cells were harvested and cell lysates were co-immunoprecipitated with antibodies specific for GFP. Co-immunoprecipitated proteins were then immunoblotted with anti-VSV or anti-GFP. Ig heavy chain indicates immunoglobulin heavy chain.

NOTCH signal strength is thought to be tightly regulated by proteins such as Numb, Deltex1, and several E3 ubiquitin ligases, as well as through intra-membrane NOTCH-receptor proteolysis after binding with ligand(s) expressed on neighboring cells (37–39). The intracellular portion of NOTCH1 contains two protein–protein interaction domains, the RAM domain, and the ankyrin (ANK) repeats, two NLS, a trans-activation domain (TAD) which is important in NOTCH1- and NOTCH2-regulated transcription but is absent from NOTCH3 and NOTCH4, and a PEST sequence, important for regulating NOTCH degradation (15). NOTCH down-stream effects are governed by binding and modulating proteins, and may be mediated by specific domains of NOTCH1. We found that only when NOTCH1 was retained in the cytoplasm could it bind to CARMA1 (Figure 4D). To define the sites of interaction between NOTCH1 and CARMA1, we obtained fluorescent fusion chimeras of NOTCH1 (GFP–N1IC), mutated the constructs, and verified their cellular localization in 293T cells (Figure 6A). Adding an additional NES sequence to GFP–N1IC mutants promoted their cytosolic retention (Figure 6A), compared to N1IC mutants lacking the additional NES. To identify those domain(s) of NOTCH1 required for its interaction with CARMA1, we co-transfected these mutants together with VSV-tagged WT CARMA1, into 293T cells and co-immunoprecipitated with antibodies specific for GFP. We detected interaction between CARMA1 and all N1IC–NES mutants except N1ICΔRAM–NES, which showed strongly reduced binding, even though its expression was restricted to the cytosol (Figure 6B). These data demonstrate that the N-terminal region of N1IC, including the RAM domain, is required for the interaction of N1IC with CARMA1, and this association likely occurs in the cytoplasm.

### Cytosolic NOTCH1 interacts with CARMA1 to enhance transcriptional activity of NF-κB

**Figure 7:**
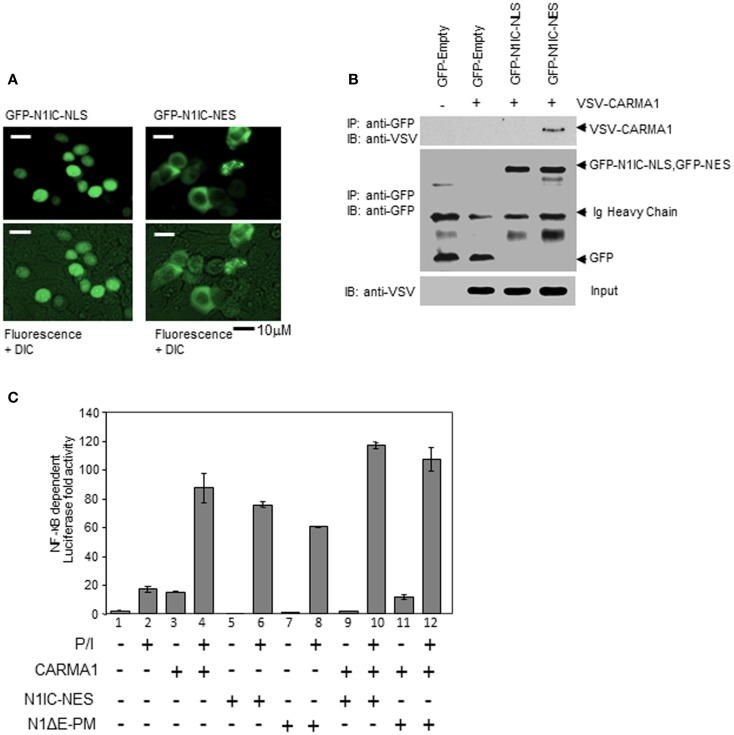
**NOTCH1 increases NF-κB activity through its direct interaction with CARMA1 in the cytoplasm**. **(A)** 293T cells were transiently transfected with GFP-N1IC–NLS or GFP-N1IC–NES and sub-cellular localization was determined using fluorescence microscopy. **(B)** CARMA1 expression plasmids were transiently co-transfected with GFP empty vector as a control, or with GFP-N1IC–NLS or GFP-N1IC–NES in 293T cells. Cytoplasmic extracts from transfected cells were co-immunoprecipitated with anti-GFP. 1/100 of input (shown) was immunoblotted with anti-VSV and anti-GFP. **(C)** Vectors expressing cytosolic N1IC–NES, membrane-tethered N1ΔE–PM, and/or cytosolic CARMA1 were co-transfected with an NF-κB luciferase reporter construct into Jurkat T cells, stimulated with PMA and CaI for 3 h, then subjected to a luciferase reporter gene assay. Ig heavy chain indicates immunoglobulin heavy chain. Data are mean + SEM of at least three independent experiments.

Canonical signaling by N11C is mediated by its effects on transcription in cooperation with the DNA binding protein, CSL (40). Translocation of N1IC to the nucleus leads to formation of N1IC/CSL complexes on the promoters of NOTCH-regulated genes. This further facilitates recruitment of transcriptional co-activators, such as p300, and the subsequent initiation of transcription. CARMA1 and the CBM complex, however, are thought to function exclusively in the cytosol. Previous studies by other groups have highlighted the contribution of CARMA1 and BCL10 to NF-κB-dependent gene expression in Jurkat T cells following stimulation with PMA and CaI or with anti-human CD3ε and anti-human CD28 (2, 26, 41). However, an integral role for NOTCH1 in mediating this process (Figures 3–5) has not been previously described. We expressed nuclear- and cytosolic-directed N1IC constructs in 293T cells and verified their cellular distribution (Figures 7A,B). We then sought to determine how cellular localization of N1IC influences its interactions with CARMA1 to regulate NF-κB activation. We co-transfected CARMA1, cytoplasmically expressed N1IC–NES, or a membrane-tethered, non-cleavable form of N1IC (N1ΔE–PM), either individually or together, with an NF-κB luciferase reporter construct into Jurkat T cells. Cells were then left unstimulated or stimulated for 3 h with PMA and CaI. We determined NF-κB activity using a luciferase reporter gene assay. Expression of CARMA1 alone induced NF-κB reporter activity ~15-fold (Figure 7C, lane 3), consistent with previous reports. PMA and CaI stimulation further increased reporter activity an additional four to fivefold, compared to unstimulated controls (Figure 7C, lane 4). When we expressed constructs of N1IC–NES or N1ΔE–PM individually, or together with CARMA1, in the absence of stimulation we detected only basal levels of NF-κB activity (Figure 7C, lanes 5, 7, 9, 11). This activity increased in stimulated Jurkat T cells expressing either of these constructs, individually (Figure 7C, lanes 6, 8). Altogether, these results are consistent with the fact that events that occur during T cell stimulation are important for downstream NF-κB activation. However, when cytosolic N1IC–NES or membrane-tethered N1ΔE–PM was expressed together with CARMA1 and cells were stimulated with PMA and CaI, we observed an additive effect on NF-κB activity (Figure 7C, lanes 10, 12), compared to expression of either construct alone in stimulated cells. Collectively, these data suggest that cytosolic and/or membrane-bound NOTCH1 is capable of activating NF-κB at time points that precede NOTCH1 nuclear accumulation and, thus, may reveal a novel non-nuclear function for NOTCH1 soon after T cell stimulation.

## Discussion

Scaffold proteins have emerged as key orchestrators of multi-component signaling complexes in many systems, including immune cells (42, 43). They function as central organizers to control a series of signal transduction cascades and mediate complex assembly by coordinating protein–protein interactions. NOTCH cell surface receptors are highly conserved type I transmembrane proteins. The N1IC intracellular fragment is comprised of domains that mediate protein–protein interaction, transcriptional activation, and proteolytic degradation. Ligand binding induces a series of enzymatic cleavages to generate N1IC allowing it to translocate to the nucleus. A large body of data describes its essential role in the transcription of target genes. Recent new studies point to novel contributions of N1IC in mediating regulatory T cell survival, a function that did not require its nuclear redistribution (20–22). Here, we utilized microcopy and biochemical approaches to demonstrate a cytoplasmic role for N1IC during the first few hours following T cell activation. In stimulated T cells, N1IC associated with members of the CBM complex, a cytosolic supramolecular structure that relays signals from the TCR. Loss of NOTCH1 expression prevented CBM assembly, abrogated IκBα phosphorylation, and diminished NF-κB binding at early time points. These observations are consistent with what is known about the requirement for the CBM signalosome upstream of classical NF-κB activation. In earlier reports, we utilized an GSI to prevent NOTCH cleavage and translocation, and showed it blocked NF-κB nuclear accumulation at the later (12 h post-stimulation) but not the earlier (first 6 h post-stimulation) time points (19). Surprisingly, in this study, we found strong NF-κB transcriptional activity within 3 h of T cell stimulation when N1IC was expressed in a form that confined it to the cytosol, or even when it was tethered to the membrane. Altogether, these data support a model whereby N1IC acts to nucleate the CBM complex and initiate NF-κB signaling downstream of TCR engagement, a cytosolic function which has not previously been described for N1IC. In light of these new data, we offer a refined interpretation of our earlier observations (19). We propose that non-nuclear N1IC may aid in initiating NF-κB signaling in stimulated T cells, but cleaved, nuclear-residing N1IC is required to sustain NF-κB activity at later time points. This two-tiered signaling model would provide a self-reinforcing feedback loop to ensure optimal NF-κB activation.

Earlier studies have identified PKCθ as necessary for initiating the signaling cascade that culminates in classical NF-κB activation (14). Within the context of the present study, therefore, we sought to ask two major questions. We were interested in knowing whether NOTCH1 acts in the same functional pathway as PKCθ and, if so, could we identify spatial and temporal events that defined their mutual regulation of NF-κB activation?

To answer the first question, we employed a murine model of skin allografting, which requires inhibition both of classical and non-classical NF-κB signaling in recipient mice to completely attenuate graft rejection (27). In the absence of the p50 subunit of NF-κB, which requires intact PKCθ signaling to translocate to the nucleus (44), we noted delayed, but not completely inhibited, graft rejection. Furthermore, when we transferred skin from BALB/c donors to recipient mice which lacked PKCθ, NOTCH1, or both, the kinetics of graft rejection were identical to those seen in the p50^null^ recipient. Altogether, these observations suggested to us that PKCθ and NOTCH1 were both acting in the same functional pathway in T cells in a mouse model of allograft rejection, and loss of either or both of these proteins produced the same rejection kinetics as loss of p50. Functional interaction between NOTCH receptors and PKC family members has been previously noted. NOTCH3IC and PKCθ were shown to cooperate to increase NF-κB signaling in thymocytes, leading to leukemogenesis (45). Although direct interaction between NOTCH3IC and PKCθ was not demonstrated in that study, NOTCH3IC expression enhanced membrane translocation of PKCθ, which also required expression of pre-TCR, and expression of all of these components were required to initiate NF-κB signaling, as measured by the nuclear translocation of NF-κB subunits.

Upon TCR engagement, CARMA1 is redistributed into lipid rafts and recruited to the IS, along with BCL10 *via* CARD–CARD interaction (3). Previous studies using amino acid substitution revealed those domains of CARMA1 that are capable of mediating protein–protein interactions and, thus, defined it as a scaffold protein (46). The SH3 domain of CARMA1 is important for its recruitment into lipid rafts, where it may function to further recruit signaling molecules into supramolecular structures targeted to the IS. We found that NOTCH1 also accumulated in lipid rafts in activated T cells. This is consistent with reports that, following T cell stimulation, endogenous NOTCH1 directly associates with proteins enriched in lipid rafts, such as p56^Lck^, CD4, and PI3K, both in Jurkat T cells and in *in vitro*-activated splenic T cells (31, 47). We observed that NOTCH1 physically associated with CARMA1 and BCL10 following T cell stimulation. Using bifluorescence complementation, we further confirmed that CARMA1 and NOTCH1 are capable of physically interacting in the cytosol.

NOTCH1-deficient cells failed to form a CBM complex, a phenomenon that, to the best of our knowledge, has not previously been described. NOTCH1 deficiency did not alter endogenous levels of CARMA1 or BCL10, thus it is unlikely that faulty CBM complex assembly in the absence of NOTCH was due to reduced expression either of CARMA1 or BCL10. A critical step in the formation of the CBM complex is phosphorylation both of CARMA1 and BCL10. How NOTCH1 may influence phosphorylation of either or both proteins remains to be investigated. It has been reported that CARMA1 is inducibly phosphorylated by PKCθ (1, 11, 48), and we did not observe physical association of PKCθ either with CARMA1 or BCL10 in the absence of NOTCH1. It has been hypothesized that CARMA1 is tethered to the membrane by an unknown protein (1). Although not the focus of this study, it is intriguing to speculate that NOTCH1 may serve this function. If NOTCH1 and CARMA1 exist in preformed complexes in unstimulated T cells, their recruitment into the IS following T cell stimulation would bring them both into close proximity to PKCθ, perhaps stabilizing the interaction between PKCθ and CARMA1, and facilitating PKCθ phosphorylation of CARMA1. This model is consistent with our observations that NOTCH1 is necessary for CBM complex assembly, as is T cell stimulation, although additional experiments are necessary to confirm this hypothesis.

To identify the critical domains of NOTCH1 required for mediating direct interaction with CARMA1, we fused additional NES sequences to N1IC mutants (19, 25) in order to redirect these proteins to the cytoplasm. We chose this approach rather than mutating the NLS sites of N1IC, since it has been reported that neither mutation nor deletion of the two NLS domains in N1IC completely abolishes nuclear distribution (49). This concurs with early studies showing the ANK repeat region also has some intrinsic capacity for nuclear localization (25, 49, 50). Of the N1IC mutants we tested, only the N1ICΔRAM construct diminished CARMA1 binding when it was exported to the cytoplasm using an NES tag. Intriguingly, although the N-terminal region of N1IC has been shown to mediate its interaction with RBP-Jκ or NF-κB (19, 50), here it is also unexpectedly responsible for its interaction with CARMA1. Additional studies will be needed to define the region(s) of CARMA1 that are required for its association with N1IC.

N1IC binding to other cytosolic proteins have also been described. N1IC was shown to interact with and inhibit the actions of JNK-interacting protein 1 (JIP1) in a variety of cell types (51). This interaction required gamma-secretase-mediated cleavage and physical association with the scaffold protein, JIP1, to negatively regulate JNK-mediated apoptosis, following glucose deprivation (51). Additionally, *via* its ANK 2 and 3 domains, N1IC can interact with non-phosphorylated apoptosis signal-regulating kinase 1 (ASK1) in the cytosol, as well as with the phosphorylated form of ASK1 in the nucleus (52). This association in MCF7 cells is thought to provide protection from apoptosis under conditions of oxidative stress (52). Thus, there may well-exist more global functions of cytosolic NOTCH1 than has been appreciated to date.

The findings presented here, that cytosolic N1IC can directly bind to CARMA1 and is required for further recruitment of BCL10 and assembly of proteins required for IKK activation, and IκBα phosphorylation strongly support a cytoplasmic function of NOTCH1 in the first few hours following TCR engagement. Together, these studies reveal the potential for a complementary and reinforcing signaling cascade in stimulated T cells, one in which N1IC acts in the cytoplasm as a molecular scaffold early on to initiate NF-κB activation and, at later time points, in the nucleus to facilitate NF-κB-mediated gene transcription.

## Author Contributions

Hyun Mu Shin, Mulualem E. Tilahun, Ok Hyun Cho, Karthik Chandiran, Christina Arieta Kuksin, Shilpa Keerthivasan performed experiments and collected and analyzed data. Abdul H. Fauq synthesized GSI. Barbara A. Osborne and Lisa M. Minter designed and supervised the experiments with contributions from Todd E. Golde and Lucio Miele. Hyun Mu Shin co-wrote the manuscript, with critical input from Margot Thome, together with Barbara A. Osborne and Lisa M. Minter.

## Conflict of Interest Statement

The authors declare that the research was conducted in the absence of any commercial or financial relationships that could be construed as a potential conflict of interest.

## Supplementary Material

The Supplementary Material for this article can be found online at http://www.frontiersin.org/Journal/10.3389/fimmu.2014.00249/abstract
