# Supplementary material for: NOTCH1 Can Initiate NF-κB Activation via Cytosolic Interactions with Components of the T Cell Signalosome
Source: Front Immunol. 2014 May 26;5:249. doi: 10.3389/fimmu.2014.00249 (PMC4033603; doi:10.3389/fimmu.2014.00249)
Supplement: Supplementary file 1 [file Presentation_1.PDF]

SUPPLEMENTARY MATERIAL

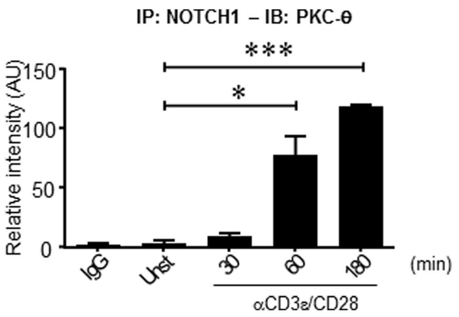

FIGURE S1 | Intensity of PKCθ bound to NOTCH1 in activated T cells increases over time. The intensity of PKCθ bound to NOTCH1, as revealed by co-immunoprecipitation, was quantified using ImageJ software. Bands representing PKCθ (**Figure 2B**, upper panel) were normalized to bands representing PKCθ input (**Figure 2B**, middle panel), for the various conditions indicated. Data represent the mean + SEM of three independent experiments. Statistical significance was determined using an unpaired Student's *t* test with two-tailed *P* value (*$P < 0.05$; **$P < 0.005$; ***$P < 0.0005$); ns, not significant.

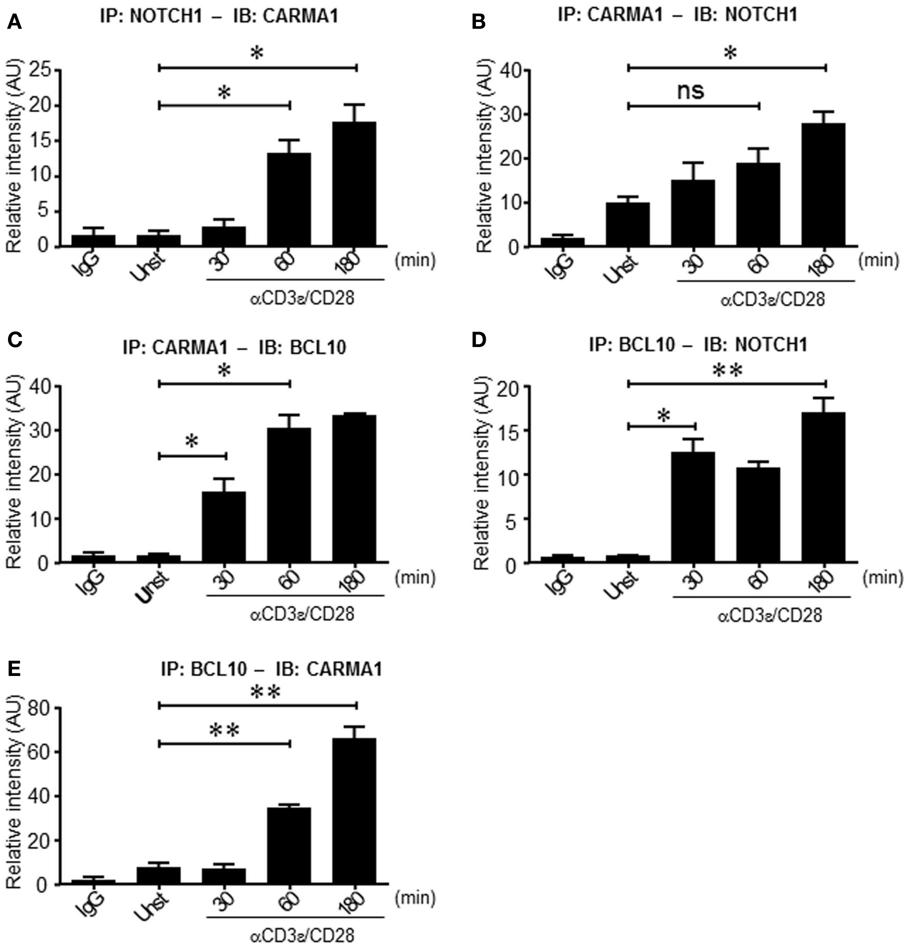

FIGURE S2 | Intensity of CBM components bound to NOTCH1 in activated T cells increases over time. The intensity of various components of the CBM complex bound to NOTCH1, or to each other, as revealed by co-immunoprecipitation, was quantified using ImageJ software. **(A)** Bands representing CARMA1 bound to NOTCH1 (**Figure 3B**, top panel) were normalized to bands representing CARMA1 input (**Figure 3B**, uppermost "input" panel), for the various conditions indicated. **(B)** Bands representing NOTCH1 bound to CARMA1 (**Figure 3B**, second panel from top) were normalized to bands representing NOTCH11 input (**Figure 3B**, lowermost "input" panel), for the various conditions indicated. **(C)** Bands representing BCL10 bound to CARMA1 (**Figure 3B**, third panel from top) were normalized to bands representing BCL10 input (**Figure 3B**, middle "input" panel), for the various conditions indicated. **(D)** Bands representing NOTCH1 bound to BCL10 (**Figure 3B**, fourth panel from top) were normalized to bands representing NOTCH1 input (**Figure 3B**, lowermost "input" panel), for the various conditions indicated. **(E)** Bands representing CARMA1 bound to BCL10 (**Figure 3B**, fifth panel from top) were normalized to bands representing CARMA1 input (**Figure 3B**, uppermost "input" panel), for the various conditions indicated. Data represent the mean + SEM of three independent experiments. Statistical significance was determined using an unpaired Student's t test with two-tailed P value ($*P < 0.05$; $**P < 0.005$; $***P < 0.0005$); ns, not significant.

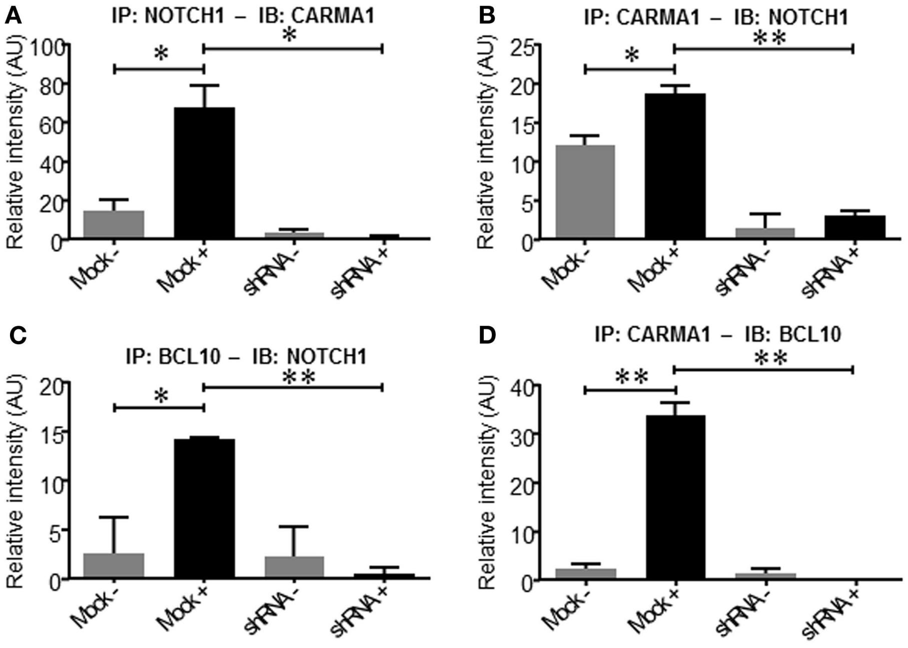

FIGURE S3 | Association of CBM components with NOTCH1 is abrogated in activated T cells when NOTCH1 expression is inhibited. The intensity of various components of the CBM bound to NOTCH1, or to each other, as revealed by co-immunoprecipitation, was quantified using ImageJ software. **(A)** Bands representing CARMA1 bound to NOTCH1 in the presence or absence of NOTCH1 (**Figure 5B**, top panel) were normalized to bands representing CARMA1 input (**Figure 5C**, third "input" panel from top), for the various conditions indicated. **(B)** Bands representing NOTCH1 bound to CARMA1 in the presence or absence of NOTCH1 (**Figure 5B**, second panel from top) were normalized to bands representing NOTCH1 input (**Figure 5C**, uppermost "input" panel), for the various conditions indicated. **(C)** Bands representing NOTCH1 bound to BCL10 in the presence or absence of NOTCH1 (**Figure 5B**, third panel from top) were normalized to bands representing NOTCH1 input (**Figure 5C**, uppermost "input" panel), for the various conditions indicated. **(D)** Bands representing BCL10 bound to CARMA1 in the presence or absence of NOTCH1 (**Figure 5B**, bottom panel) were normalized to bands representing CARMA1 input (**Figure 5C**, third "input" panel from top), for the various conditions indicated. For all panels: Mock-infected, without stimulation: Mock−; Mock-infected with stimulation: Mock+; shRNA to NOTCH1-infected without stimulation: shRNA−; shRNA to NOTCH1-infected with stimulation: shRNA−. Data represent the mean + SEM of three independent experiments. Statistical significance was determined using an unpaired Student's *t* test with two-tailed *P* value (*$P < 0.05$; **$P < 0.005$; ***$P < 0.0005$); ns, not significant.

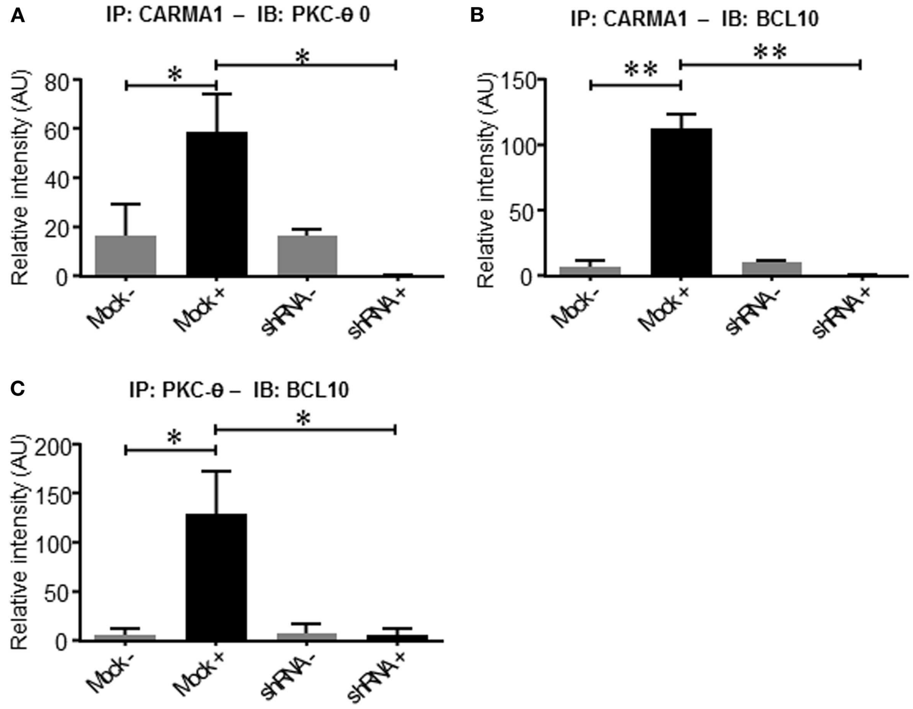

FIGURE S4 | Association of CBM components with each other is abrogated in activated T cells when NOTCH1 expression is inhibited. The intensity of various components of the CBM complex bound to each other in the presence or absence of NOTCH1, as revealed by co-immunoprecipitation, was quantified using ImageJ software. **(A)** Bands representing PKCθ bound to CARMA1 in the presence or absence of NOTCH1 (**Figure 5C**, top panel) were normalized to bands representing PKCθ input (**Figure 5C**, lowermost "input" panel), for the various conditions indicated. **(B)** Bands representing BCL10 bound to CARMA1 in the presence or absence of NOTCH1 (**Figure 5C**, top panel) were normalized to bands representing CARMA1 input (**Figure 5C**, third "input" panel from top), for the various conditions indicated. **(C)** Bands representing BCL10 bound to PKCθ in the presence or absence of NOTCH1 (**Figure 5C**, top panel) were normalized to bands BCL10 representing input (**Figure 5C**, second "input" panel from top), for the various conditions indicated. For all panels: Mock-infected, without stimulation: Mock−; Mock-infected with stimulation: Mock+; shRNA to NOTCH1-infected without stimulation: shRNA−; shRNA to NOTCH1-infected with stimulation: shRNA−. Data represent the mean + SEM of three independent experiments. Statistical significance was determined using an unpaired Student's t test with two-tailed P value ($*P < 0.05$; $**P < 0.005$; $***P < 0.0005$); ns, not significant.

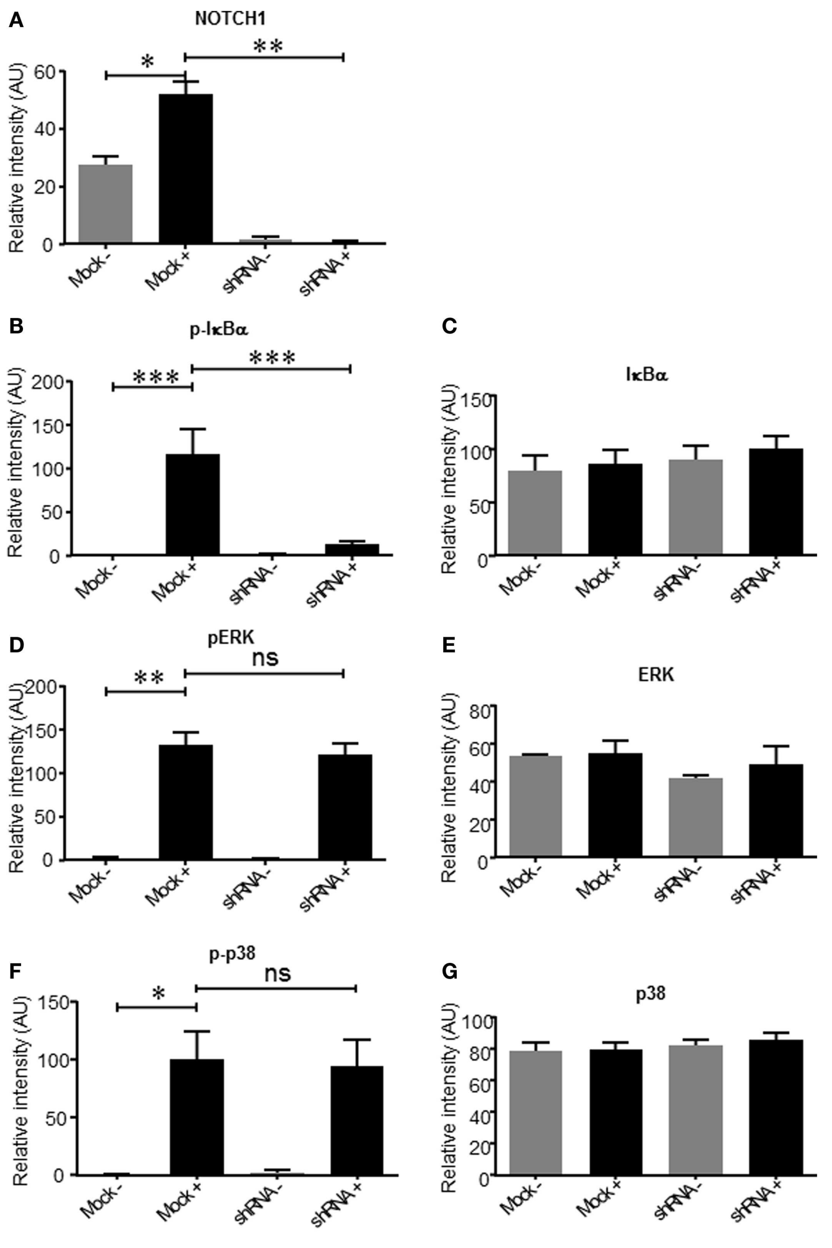

FIGURE S5 | Inhibiting NOTCH1 expression differentially affects downstream signaling pathways. The intensity of various proteins important for T cell activation expressed in the presence or absence of NOTCH1, as revealed by SDS PAGE and immunoblotting, was quantified using ImageJ software. **(A)** Bands representing NOTCH1 expression following mock infection or infection with shRNA to NOTCH1 in unstimulated and stimulated T cells (**Figure 5D**, uppermost panel), were normalized to input of β-actin (blot not shown). **(B)** Bands representing phosphorylated IkBa expression following mock infection or infection with shRNA to NOTCH1 in unstimulated and stimulated T cells (**Figure 5D**, second panel from top), were normalized to input of total IkBa (**Figure 5D**, third panel from top). **(C)** Bands representing total IkBa expression following mock infection or infection with shRNA to NOTCH1 in unstimulated and stimulated T cells (**Figure 5D**, third panel from top), were normalized to input of β-actin (blot not shown). **(D)** Bands representing phosphorylated ERK expression following mock infection or infection with shRNA to NOTCH1 in unstimulated and stimulated T cells (**Figure 5D**, fourth panel from top), were normalized to input of total ERK (**Figure 5D**, third panel from bottom). **(E)** Bands representing total ERK expression following mock infection or infection with shRNA to NOTCH1 in unstimulated and stimulated T cells (**Figure 5D**, third panel from bottom), were normalized to input of β-actin (blot not shown). **(F)** Bands representing phosphorylated p38 expression following mock infection or infection with shRNA to NOTCH1 in unstimulated and stimulated T cells (**Figure 5D**, second panel from bottom), were normalized to input of total p38 (**Figure 5D**, lowermost panel). **(G)** Bands representing total p38 expression following mock infection or infection with shRNA to NOTCH1 in unstimulated and stimulated T cells (**Figure 5D**, lowermost panel), were normalized to input of β-actin (blot not shown). For all panels: Mock-infected, without stimulation: Mock−; Mock-infected with stimulation: Mock+; shRNA to NOTCH1-infected without stimulation: shRNA−; shRNA to NOTCH1-infected with stimulation: shRNA−. Data represent the mean + SEM of three independent experiments. Statistical significance was determined using an unpaired Student's t test with two-tailed P value ($^*P < 0.05$; $^{**}P < 0.005$; $^{***}P < 0.0005$); ns, not significant.
